# Inter-generational resemblance of methylation levels at circadian genes and associations with phenology in the barn swallow

**DOI:** 10.1038/s41598-019-42798-3

**Published:** 2019-04-24

**Authors:** Nicola Saino, Benedetta Albetti, Roberto Ambrosini, Manuela Caprioli, Alessandra Costanzo, Jacopo Mariani, Marco Parolini, Andrea Romano, Diego Rubolini, Giulio Formenti, Luca Gianfranceschi, Valentina Bollati

**Affiliations:** 1 Department of Environmental Science and Policy, University of Milan, via Celoria 26, I-20133 Milan, Italy; 2 Department of Clinical Sciences and Community Health, via S. Barnaba 8, I-20122 Milan, Italy; 3 Department of Ecology and Evolution, University of Lausanne, Building Biophore, CH-1015 Lausanne, Switzerland; 4 Department of Biosciences, University of Milan, via Celoria 26, I-20133 Milan, Italy

**Keywords:** Ecological genetics, Behavioural ecology

## Abstract

Regulation of gene expression can occur via epigenetic effects as mediated by DNA methylation. The potential for epigenetic effects to be transmitted across generations, thus modulating phenotypic variation and affecting ecological and evolutionary processes, is increasingly appreciated. However, the study of variation in epigenomes and inter-generational transmission of epigenetic alterations in wild populations is at its very infancy. We studied sex- and age-related variation in DNA methylation and parent-offspring resemblance in methylation profiles in the barn swallows. We focused on a class of highly conserved ‘clock’ genes (clock, cry1, per2, per3, timeless) relevant in the timing of activities of major ecological importance. In addition, we considerably expanded previous analyses on the relationship between methylation at clock genes and breeding date, a key fitness trait in barn swallows. We found positive assortative mating for methylation at one clock *locus*. Methylation varied between the nestling and the adult stage, and according to sex. Individuals with relatively high methylation as nestlings also had high methylation levels when adults. Extensive parent-nestling resemblance in methylation levels was observed. Occurrence of extra-pair fertilizations allowed to disclose evidence hinting at a prevalence of paternal germline or sperm quality effects over common environment effects in generating father-offspring resemblance in methylation. Finally, we found an association between methylation at the clock poly-Q region, but not at other *loci*, and breeding date. We thus provided evidence for sex-dependent variation and the first account of parent-offspring resemblance in methylation in any wild vertebrate. We also showed that epigenetics may influence phenotypic plasticity of timing of life cycle events, thus having a major impact on fitness.

## Introduction

Environmental conditions can induce regulation of the expression and function of genes via epigenetic effects, without directly altering nucleotide sequence^1–3^. Epigenetic programming can produce differential gene expression among individuals, thus generating phenotypic differences even from the same genetic substrate^2,4–7^.

Epigenetic alterations can occur in response to a wide variety of extrinsic environmental *stimuli* ranging from nutritional conditions to social and chemical stress^3,7,8^. Epigenetic alterations, in turn, are heritable^3,9–12^ and thus have the potential to result in transmission of new phenotypes across multiple generations. The possibility that ancestors’ experiences result in epigenetic transmission across generation borders of phenotypic traits has obvious, tremendous and yet poorly explored implications for ecological and evolutionary processes^2,7^. Indeed, heritable epigenetic effects are known to affect phenotypic variation at major life-history traits including physiology, morphology and behavior^3,9,13,14^.

Epigenetic modulation of gene expression occurs through major classes of mechanisms that include DNA methylation, histone modifications, the effect of non-coding RNAs, and the activity of chaperones^3,7,15,16^. DNA methylation, particularly at the palindrome dinucleotide sequences 5′CpG3′ at the 5 position of cytosine, is generally regarded as the most common epigenetic mark in mammals, although information for other vertebrates is scanty. Methylation of cytosines located in gene promoters modulates gene transcription, is involved in alternative promoter usage and regulation of enhancer activity, and often causes downregulation of the gene^9,15,17,18^. Epigenetic effects mediated by DNA methylation may be a key mechanism that controls variation in behavioral traits^13,18–21^. For example, methylation at the dopamine receptor D4 (DRD4) gene is related to exploratory behavior^22^, while methylation at the dopamine receptor and serotonin transporter is related to novelty seeking behaviour in great tits (*Parus major*)^21^. In addition, methylation at the agouti-related peptide is significantly related to sexual coloration in the black grouse (*Lyrurus tetrix*)^23^.

DNA methylation can also be transmitted across generations, generally meaning that offspring resemble their parents in methylation levels, as shown exclusively by laboratory studies^9,11,12^. Such inheritance of methylation states can arise via different pathways. First, ‘true’ transgenerational inheritance occurs via the germline if methylation profile of the gametes is carried over to the zygote and later developmental stages. Rigorously demonstrating ‘true’ transgenerational inheritance of methylation profiles is extremely difficult, particularly in the wild, as it requires showing that ancestors resemble their F3 or later-generation descendants. Despite these difficulties, some studies have provided evidence for transgenerational inheritance of responses to parental experiences including diet, stressful conditions and exposure to toxins^7,9^, and via gamete-mediated inheritance of methylation marks. Such transmission by germline methylation has been demonstrated in studies of the effects of vinclozolin in mice on anxiety-like behaviors^24,25^ and also in a single study of birds under laboratory conditions^26^. However, it must be emphasized that while environmentally driven changes in DNA methylation has been repeatedly documented to be inherited in plants, the evidence for transgenerational environmental effects on methylation is still scanty and contentious for taxa like vertebrates^27^.

An alternative pathway of methylation inheritance is via ‘soma-to-soma’ interactions. These can occur when the prenatal environment in terms for example of in utero conditions or egg composition directly affect the methylome of the developing offspring^7,16^. In addition, RNA molecules stored in sperm and components of the seminal fluid also have the potential for transgenerational transmission of epigenetic traits^9,15,28^. Such soma-to-soma effects can also occur via general environmental effects whenever ancestors’ experience of environmental conditions causes a new epigenetic mark and phenotype to arise^29,30^ and ancestors tend to reinstate similar environmental (e.g. physical or social) conditions across subsequent generations, causing the same epigenetic mark to re-appear in descendants^9^. Finally, parent offspring resemblance may arise because of transgenerational genetic effects, whereby similarity in methylation levels in parents and offspring arises because of similarity in their genetic background^7,31^.

Inheritance of methylation may not necessarily occur symmetrically from either parental genome, as exemplified at a paradigmatic extreme by parental sex-specific genetic imprinting^32^. The evidence for the relative contribution of maternal versus paternal methylation states to descendant methylation patterns in humans and other vertebrates is mixed and parental sex-specific inheritance effects may vary among taxa^3,33–35^.

It is worth emphasizing that, independently of the exact mechanisms that determine resemblance in methylation states across generations, any such resemblance has extremely important ecological and evolutionary consequences because it implies that new epigenetic phenotypes can be carried over to subsequent generations independently of genetic inheritance. Yet, to the best of our knowledge, no study has been published to date on any vertebrate species in the wild where resemblance in methylation at the single-*locus* level between parent and offspring has been investigated.

The first main aim of this study thus was to investigate whether offspring, at their nestling stage and also once they attain sexual maturity, resemble their parents in CpG methylation at five highly conserved genes (clock, per2, per3, cry1, timeless), using a small, migratory bird, the barn swallow (*Hirundo rustica*) as a study system. In doing so, we also tested for any differential effect of methylation state of either parent on methylation of offspring of either sex, and on age- (nestling vs adult) and sex-specific variation in methylation^21,36^. Stemming from current interest on control of timing of major life cycle stages in the barn swallow^37^, in the present study we focused on methylation at ‘clock’ genes whose products dynamically interact to elicit rhythmic patterns of transcription, translation, biochemical and physiological processes, and behavior^38,39^ and are thus involved in circadian rhythmicity and photoperiodic responses (see also Supplementary Material). In addition, we tested for resemblance in methylation levels between pair members because similarity in methylation profiles at photoperiodic genes might influence mate choice or, reciprocally, synchronization in reproductive behavior might result in resemblance in methylation levels between pair members.

To our goals, we measured methylation at the focal *loci* in blood cells of 65 barn swallows (from 58 social pairs) when they were nestlings and when they were subsequently recruited as 1-year-old sexually mature adults, and in their parents. Because nucleated erythrocytes in barn swallows and other birds represent a large fraction of nucleated blood cells^40^, our methylation data essentially reflect methylation of erythrocytes. Parent-offspring resemblance in methylation can result from diverse mechanisms, which are extremely difficult to tease apart (see above), especially in the wild. When extra-pair fertilizations occur, however, two types of families are naturally established: first, families where parental fathers (i.e. the males that attend the offspring) are also the genetic fathers of the offspring (hereafter, within-pair offspring, WPO) that they attend; second, families where the parental father is the ‘social’, but not the genetic father (hereafter, ‘social father’) of one or more of the offspring (hereafter, extra-pair offspring, EPO) that they attend. Extra-pair fertilizations, which are common in the barn swallow^41,42^, afford an opportunity to get an insight into the contribution of different mechanisms of parent-offspring resemblance in methylation states, and we therefore subjected the 65 sampled offspring to genetic parentage tests. If genetic father-WPO resemblance in methylation is stronger than social father-EPO resemblance, a mechanism of inheritance mediated by the paternal germline or via non-genetic sperm and seminal fluid constituents can be invoked. Because the reciprocal condition whereby mothers attend non-genetically related offspring in their nest is very uncommon in barn swallows^43–45^, as in the majority of altricial birds^46^, no such approach could be undertaken to assess the relative contribution of maternal germline/egg effects as compared to postnatal environmental effects.

Very limited background knowledge on methylation in wild birds, however, prevented us from formulating explicit predictions on the differential father-offspring relationships according to paternity and on sex- and age-dependency of methylation levels.

In a previous study focusing on two clock *loci* (clock poly-Q and clock 5′-UTR) we showed that methylation at the clock poly-Q was statistically associated with spring migration and breeding date in the same barn swallow population^37^. The previous analysis was restricted to relatively old (two or more years) individuals and focused on just two *loci*. Here, we expand the analysis of the association between methylation at these two *loci* and breeding date, which is a major fitness trait because in barn swallows it predicts both seasonal reproductive success and offspring quality^37,42,47,48^, on a much larger sample of birds and we also assess whether methylation at other genes besides clock is related to breeding date. The importance of such replication studies to test for consistency of results has been repeatedly advocated^49^. In the analyses of breeding date in relation to methylation at clock 5′-UTR and clock poly-Q, we thus also considered the data included in Saino *et al*.^37^ (see also below).

## Results

### Methylation in pair members

**Figure 1:**
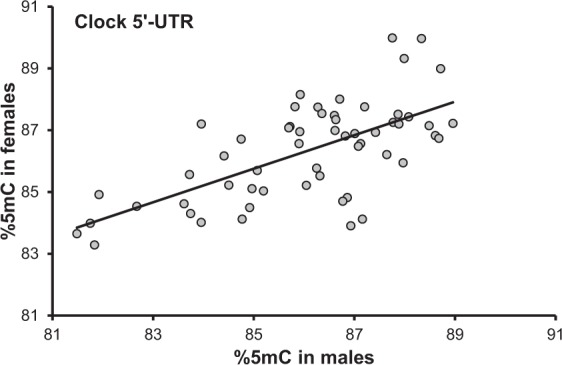
Relationship between methylation at clock 5′-UTR in females and in their male mates.

LMMs showed that methylation of males at clock 5′-UTR significantly and positively predicted methylation of their female mate (F_1,39_ = 14.75, P < 0.001^†^, coefficient: 0.310 (0.081); Fig. 1) whereas the relationships between mates at the other *loci* were non-significant (Table 1S).

**Table 1:** Methylation in relation to life stage and sex. Linear mixed models of methylation at the six *loci* in relation to life stage (adult vs nestling) and sex. In the models we included year, colony and family as random effects. The interaction term between life stage and sex was removed when its effect was statistically non-significant before FDR. Thus, for example, the main effects of life stage and sex on clock poly-Q methylation are computed while excluding from the model the interaction effect, as indicated by the asterisk. See Fig. 2 for mean within-group values. n = sample size.

	F	df	P
Clock 5′-UTR (n = 245)			
Life stage	19.81	1,183	<0.001^†^
Sex	4.53	1,183	0.035
Life stage × Sex^a^	7.82	1,183	0.006^†^
Clock poly-Q (n = 242)			
Life stage	19.00	1,182	<0.001^†^
Sex	24.14	1,182	<0.001^†^
Life stage × Sex*	3.92	1,181	0.049
Cry1 (n = 229)			
Life stage	2.50	1,169	0.116
Sex	2.46	1,169	0.118
Life stage × Sex*	0.00	1,168	0.997
Per2 (n = 238)			
Life stage	0.35	1,178	0.514
Sex	0.74	1,178	0.392
Life stage × Sex*	0.12	1,177	0.728
Per3 (n = 243)			
Life stage	4.32	1,183	0.039
Sex	2.99	1,183	0.085
Life stage × Sex*	0.37	1,182	0.542
Timeless (n = 191)			
Life stage	38.84	1,131	<0.001^†^
Sex	15.99	1,131	0.001^†^
Life stage × Sex*	0.15	1,130	0.697

^a^Adult females > nestling females; nestling males > nestling females at post hoc tests (P < 0.009); *Interaction term excluded from the model including main effects.

^†^Indicates statistical significance after FDR correction; the P-values reported in the table are those estimated before FDR correction.

### Methylation in relation to sex and life stage

**Figure 2:**
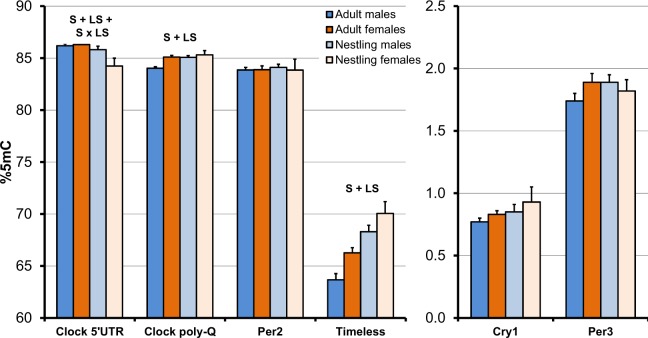
Mean (+SE) methylation at six *loci* of adult and nestling male and female barn swallows from 58 families. Means for the adult life stage are computed by pooling parents with their offspring considered at the adult (1-year old) stage. S: significant difference according to sex; LS: significant difference according to life stage (adult vs nestling); S × LS: significant effect of the sex by life stage interaction. See also Table 1.

LMMs with sex and life stage as fixed-effect factors showed a composite pattern of variation in methylation at the six *loci* (Table 1; Fig. 2). Methylation at clock 5′-UTR was lower in females than in males and in nestlings compared to adults (Table 1; Fig. 2). However, the sex-difference also depended on life stage, as shown by the significant two way-interaction (Table 1; Fig. 2). Specifically, there was a significant difference between nestling and adult females but not males and, in addition, there was a significant difference between nestling (but not adult) males and females (Table 1; Fig. 2). For clock poly-Q and timeless, methylation was significantly higher in nestlings than in adults and in females compared to males (Table 1; Fig. 2). Methylation at cry1, per2 and per3 did not vary according to sex or life stage (Table 1; Fig. 2).

In these models we also tested the random effects of year of birth of the offspring and colony. At no *locus* did colony predict variation in methylation (χ^2^ < 0.3, df = 1, P > 0.65 for all *loci*). Methylation was found to significantly vary among years for clock 5′-UTR (χ^2^ = 25.0, df = 1, P < 0.001^†^), and per3 (χ^2^ = 13.1 df = 1, P < 0.001^†^), and marginally non-significantly (after FDR) so for timeless (χ ^2^ = 4.2, df = 1, P = 0.040), but not for the other *loci* (χ ^2^ < 2.1, df = 1, P > 0.14 in all cases).

### Methylation at the nestling and adult stage

**Figure 3:**
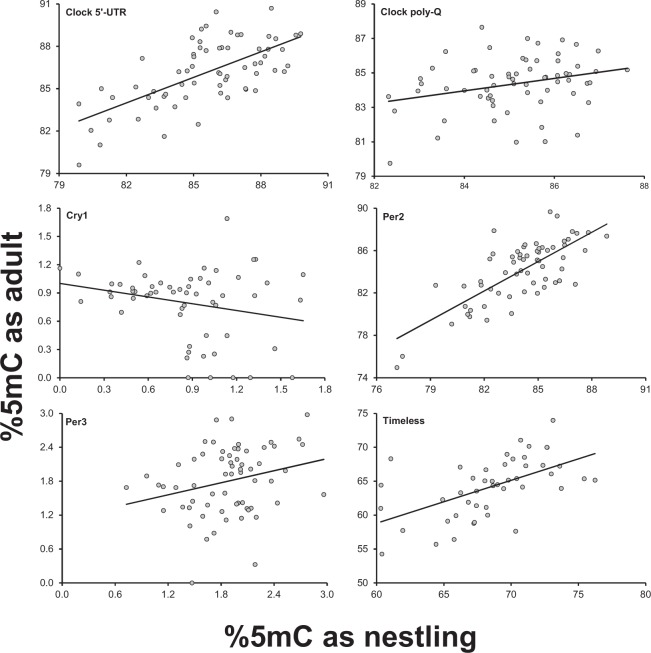
Relationships between methylation at the six *loci* of offspring at the time when they were recruited as 1-year old adults and their own methylation as nestlings.

Methylation at the nestling stage significantly and positively predicted methylation at the 1-year-old adult stage for clock 5′-UTR, clock poly-Q, per2 and timeless (Table 2; Fig. 3). The relationship was significantly negative for cry1 (Table 2; Fig. 3). Finally, no relationship was observed between the nestling and the adult stage for methylation at per3 (Table 2; Fig. 3).

**Table 2:** Relationship between methylation at the offspring adult and nestling stage. Linear mixed models of methylation at the six *loci* in the offspring at the adult (1-year old) stage in relation to their methylation at the nestling stage. Colony and year are included in all models as random effects.

	No. of offspring	F	df	P	coefficient (SE)
Clock 5′-UTR	65	6.61	1,47	0.013^†^	0.23 (0.09)
Clock poly-Q	64	4.99	1,46	0.030^†^	0.27 (0.12)
Cry1	57	11.57	1,40	0.002^†^	−2.19 (0.64)
Per2	63	73.89	1,45	<0.001^†^	0.72 (0.08)
Per3	64	0.27	1,46	0.606	0.23 (0.43)
Timeless	46	19.78	1.29	0.001^†^	0.38 (0.08)

^†^Indicates statistical significance after FDR correction; the P-values reported in the table are those estimated before FDR correction.

### Methylation in parents and offspring

**Figure 4:**
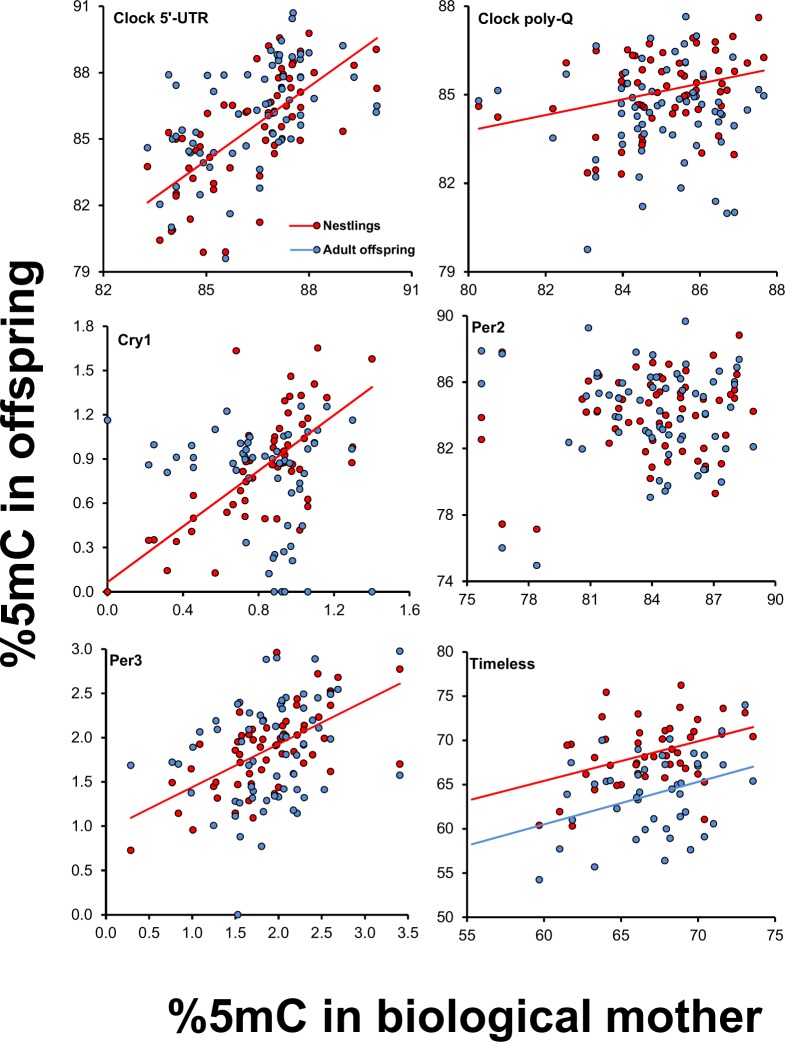
Relationships between offspring methylation at the nestling or 1-year-old adult stage and methylation of their mother. Statistically significant relationships are shown.

Methylation of nestlings at clock 5′-UTR, clock poly-Q, cry1, per3 and timeless significantly increased with methylation of their genetic mother (Table 3; Fig. 4). At the adult offspring stage, however, a significant positive relationship with maternal methylation persisted only for timeless (Table 3; Fig. 4).

**Table 3:** Relationship between methylation of the offspring and their father and mother. Linear mixed models of methylation in the offspring at the nestling or at the adult (1-year-old) stage in relation to paternity and methylation of the father (genetic or social) and of the mother. Colony and year are included in all models as random effects. Separate models were run for paternal and maternal methylation at clock 5′-UTR because of collinearity (see *Statistical analyses*). The interaction term between paternity and paternal methylation was removed from the models when its effect was statistically non-significant before FDR correction. Thus, for example, the main effects on nestling cry1 are computed while excluding from the model the interaction effect, as indicated by the asterisk. The slopes of the relationships for extra-pair (EPO) and within-pair (WPO) offspring are shown and significant ones are bolded. ‘n’ values in parentheses are the number of nestlings or, respectively, 1-year-old recruits that were included in the analyses. The maternal methylation by offspring sex interaction was retained only for per2, based on statistical significance.

	Nestling offspring				Adult offspring			
	F	df	P	Coefficient LS means (SE)	F	df	P	coefficient (SE)
Clock 5′-UTR (n = 64, 64)								
Paternity	5.00	1,43	0.031		1.26	1,45	0.267	
Sex	5.73	1,43	0.021	Males: 68.0 (0.48) Females: 67.0 (0.60)				
Paternal methylation	1.36	1,43	0.249		3.52	1,45	0.067	0.21 (0.11)
Paternity × Paternal methylation	4.91	1,43	0.032	EPO: −0.09 (0.21) WPO: **0.40 (0.12)**^**a**^	0.26*	1,44	0.613	EPO: 0.12 (0.23) WPO: 0.24 (0.15)
Clock 5′-UTR (n = 65, 65)								
Sex	8.95	1,46	0.004	−1.30 (0.43)				
Maternal methylation	15.28	1,46	<0.001^†^	0.53 (0.14)	0.01	1,47	0.944	0.01 (0.15)
Clock poly-Q (n = 61, 62)								
Paternity	13.33	1,40	<0.001		4.78	1,41	0.035	
Sex					6.06	1,41	0.018	Males: 66.7 (0.18) Females: 67.6 (0.38)
Paternal methylation	0.01	1,40	0.933		1.01	1,41	0.322	0.10 (0.10)
Maternal methylation	11.59	1,40	0.002^†^	0.26 (0.08)	0.34	1,41	0.563	−0.06 (0.10)
Paternity × Paternal methylation	13.10	1,40	<0.001^†^	EPO: **−0.27 (0.12)**^**b**^ WPO: **0.29 (0.10)**^**c**^	3.05*	1,40	0.088	EPO: −0.12 (0.16) WPO: 0.24 (0.13)
Cry1 (n = 54, 56)								
Paternity	0.18	1,35	0.676		3.67	1,36	0.063	
Paternal methylation	1.01	1,35	0.322	0.37 (0.37)	9.94	1,36	0.003^†^	1.94 (0.62)
Maternal methylation	56.99	1,35	<0.001^†^	3.74 (0.49)	2.63	1,36	0.113	−1.34 (0.83)
Paternity × Paternal methylation	0.20*	1,34	0.655	EPO: 0.72 (0.84) WPO: 0.30 (0.41)	0.16*	1,35	0.687	EPO: 2.46 (1.44) WPO: **1.82 (0.69)**^**e**^
Per2 (n = 60, 60)								
Paternity	0.92	1,38	0.345		3.06	1,38	0.088	
Sex	13.34	1,38	<0.001		14.38	1,38	<0.001	
Paternal methylation	1.95	1,38	0.171		6.34	1,38^†^	0.016^†^	0.25 (0.10)
Maternal methylation	8.74	1,38	0.005		12.65	1,38	0.001	
Sex × maternal methylation	13.37	1,38	<0.001^†^	Males: −0.03 (0.08) Females: **0.58 (0.15)**^**d**^	14.20	1,38	<0.001^†^	Males: −0.17 (0.10) Females: **0.63 (0.19)**^**f**^
Paternity × Paternal methylation	2.50*	1,37	0.122	EPO: −0.16 (0.19) WPO: 0.18(0.09)	0.17*	1,37	0.680	EPO: 0.17 (0.24) WPO: **0.28 (0.11)**^**g**^
Per3 (n = 62, 63)								
Paternity	1.68	1,42	0.202		0.00	1,43	0.970	
Paternal methylation	1.89	1,42	0.177	0.24 (0.17)	24.82	1,43	<0.001^†^	1.43 (0.29)
Maternal methylation	32.70	1,42	<0.001^†^	1.01 (0.18)	1.19	1,43	0.283	0.32 (0.29)
Paternity × Paternal methylation	2.18*	1,41	0.147	EPO: −0.23 (0.36) WPO: 0.36 (0.19)	1.01*	1,42	0.321	EPO: 0.95 (0.56) WPO: **1.57 (0.32)**^**h**^
Timeless (n = 33, 33)								
Paternity	0.18	1,14	0.674		0.09	1,14	0.769	
Paternal methylation	0.22	1,14	0.648	0.03 (0.06)	3.45	1,14	0.084	0.13 (0.07)
Maternal methylation	14.06	1,14	0.002^†^	0.38 (0.10)	12.46	1,14	0.003^†^	0.44 (0.12)
Paternity × Paternal methylation	0.09*	1,13	0.772	EPO: 0.07 (0.13) WPO: 0.03 (0.07)	0.02*	1,13	0.885	EPO: 0.11 (0.16) WPO: 0.14 (0.08)

^a^t = 3.48, P = 0.001; ^b^t = −2.30, P = 0.027; ^c^t = 2.87, P = 0.007; ^d^t = 3.93, P < 0.001; ^e^t = 2.64, P = 0.0125; ^f^t = 3.32, P = 0.002; ^g^t = 2.44, P = 0.020; ^h^t = 4.94 P < 0.001.

^†^Indicates statistical significance after FDR correction; the P-values reported in the table are those estimated before FDR correction.

*Term excluded from the model including the main effects of Paternity, Paternal methylation and Maternal methylation.

**Figure 5:**
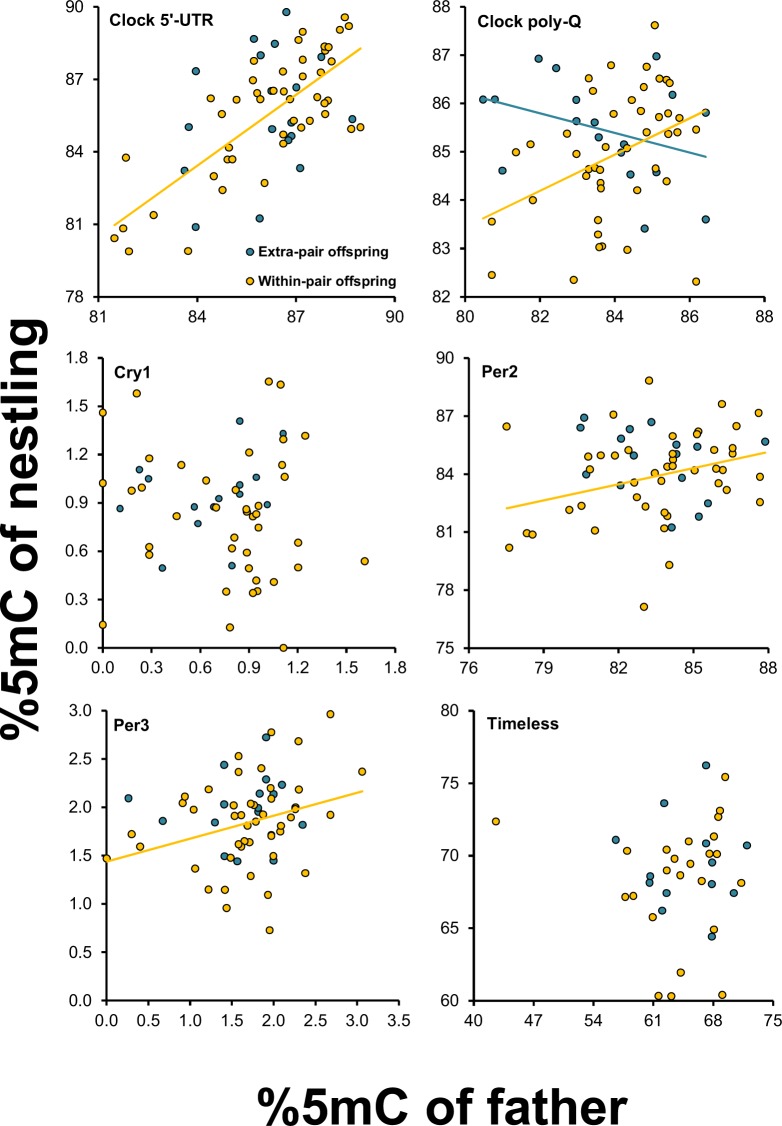
Relationships between within- or extra-pair offspring methylation at the nestling stage and methylation of the genetic or the social, non-genetic father. Statistically significant relationships are shown.

Nestling methylation differentially covaried with methylation of the genetic as compared to the social (but non-genetic) father at clock poly-Q (Table 3; Fig. 5). The effect of the paternity by paternal methylation term was either marginally non-significant (clock 5′-UTR) after FDR correction or far from significance for the other *loci* (Table 3). A closer inspection of within-paternity group coefficients (see *Statistical analyses* and SOM for a justification) showed that nestling methylation was significantly positively related to methylation of the genetic father at clock 5′-UTR and clock poly-Q, whereas the relationships for the social father were either significantly negative (clock poly-Q) or non-significant (Table 3).

At no *locus* did offspring methylation at the adult stage significantly, differentially covary with methylation of the genetic as compared to the social father (Table 3). Again, however, inspection of the within paternity group coefficients showed significant positive relationships between offspring and genetic father’s methylation at some *loci* (cry1, per2, per3) whereas no significant relationship with methylation of the social father existed at any *locus* (Table 3). Removal of the statistically non-significant paternity by paternal methylation interaction effect disclosed a significant positive relationship between adult offspring methylation and paternal (independent of genetic parentage) methylation at cry1, per2 and per3.

The effect of the interaction between maternal methylation and offspring sex significantly predicted offspring methylation at per2 at both offspring life stages (Table 3). Maternal methylation positively predicted methylation of daughters but not sons (Table 3). However, analyses restricted to the genetic fathers and their within-pair offspring did not disclose any significant interaction between methylation of the father and sex of the offspring on offspring methylation (F values associated to P values always >0.06; see also SOM).

Thus, there was a composite pattern of differential covariation between methylation in the offspring at either the nestling or the adult stage and methylation of the genetic versus the social father, with some hint for a stronger relationship between methylation in the offspring and in the genetic father as compared to the social father.

### Methylation and breeding date

**Figure 6:**
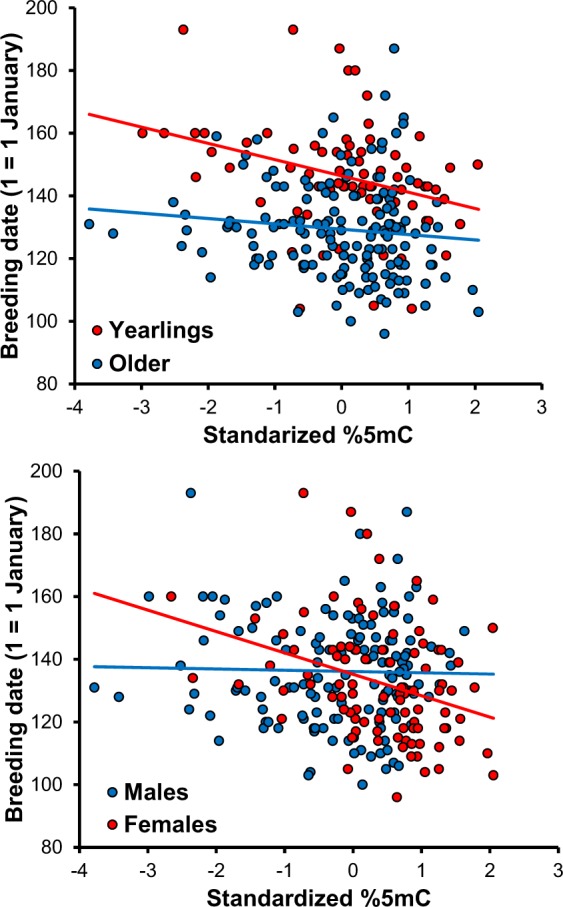
Relationship between breeding date and methylation at clock poly-Q in individuals of two age classes and according to sex. Methylation data were standardized to a mean of zero and variance of 1 within each of the two datasets (the present one and that from Saino *et al*.^37^) included in the analyses. The relationships were significantly negative within both age classes and for females but not for males.

Breeding date was significantly predicted by the interaction between methylation at clock poly-Q and sex, whereas the interaction effect between methylation at clock poly-Q and age was non-significant (Table 4; Fig. 6). In females, breeding date was significantly earlier as methylation increased (Table 4; Fig. 6). In both age classes, the relationship between breeding date and methylation was significantly negative, but it was marginally non-significantly more steep for yearlings than for older individuals (Table 4; Fig. 6).

**Table 4:** Variation in breeding date in relation to methylation at clock. Linear mixed model of breeding date in relation to methylation at clock poly-Q or clock 5′-UTR, sex and age (yearling vs older). Family, year and study area (Italy or Switzerland) were included in the models as random effects. Sample size was 234 individuals (75 yearlings, 159 older individuals; 141 males; 93 females) for clock poly-Q and 236 individuals (77, 159, 143, 93) for clock 5′-UTR.

	F	df	P	coefficient (SE)
Clock poly-Q				
Sex	0.73	1,86	0.396	
Age	40.33	1,86	<0.001	
Methylation	10.12	1,86	0.002	
Sex × age	1.35	1,86	0.249	
Age × methylation	3.44	1,86	0.067	Yearlings: −4.89 (1.54)^a^ Older: −1.45 (1.16)^b^
Sex × methylation	5.07	1,86	0.027	Males: −1.06 (1.12) Females: −5.28 (1.58)^c^
Clock 5′-UTR				
Sex	5.37	1,91	0.023	
Age	41.31	1,91	<0.001	
Methylation	3.58	1,91	0.062	−2.17 (1.15)

^a^t = −3.18, P = 0.002; ^b^t = −1.25, P = 0.215; ^c^t = −3.35, P = 0.0012.

A model excluding the non-significant interaction effects showed a marginally non-significant trend for breeding date to be earlier as methylation at clock 5′-UTR increased (Table 4).

The effect of methylation at the other *loci* did never attain statistical significance (P always >0.06).

## Discussion

The studies of DNA methylation potentially mediating epigenetic effects in wild animal populations are at their infancy, and even basic information on individual variation in methylation levels, consistency in methylation at different life stages, and inter-generational resemblance is lacking. Yet, DNA methylation and other pathways of epigenetic alterations of gene expression can have profound effects on the formation and maintenance of phenotypic variation and thus major potential, and yet largely unexplored, consequences for ecological and evolutionary processes.

In this correlational study, we contribute to start filling some of these major gaps of knowledge by focusing on a class of highly conserved genes contributing to the circadian clock system. We first analyzed age- and sex-related variation in methylation levels at six focal genetic *loci* in a wild population of barn swallow, and found variation in methylation according to age and between males and females. Second, individuals were highly consistent in relative methylation levels between the nestling and the yearling life stage at most *loci*. Third, offspring resembled their genetic mother and their attending father in methylation particularly at the nestling stage. The occurrence of extra-pair fertilizations in the barn swallows allowed to identify some hints of stronger resemblance between the offspring and their genetic father compared to the non-genetic social father, possibly suggesting inheritance of methylation levels not only mediated by common-environment effects. In addition, methylation at clock 5′-UTR was found to positively covary between breeding mates. Finally, by largely expanding a previous dataset, we confirmed that methylation at clock poly-Q negatively predicts a major phenological fitness trait, breeding date, of females but not males and we provided novel evidence that breeding date is not predicted by methylation at the other *loci*. Admittedly, in the present study we did not investigate the environmental factors that govern variation in methylation levels, which was found to occur among years (clock 5′-UTR and per3) but not among colonies. The ecological causes and the ontogenetic and sex-dependent mechanisms behind variation in methylation levels thus remain to be elucidated.

### Methylation in mates

We found evidence for non-random mating (i.e. assortative mating according to its definition in^34^) according to methylation at clock 5′-UTR but not at the other *loci*. To the best of our knowledge, this is the first evidence for positive assortative mating according to methylation profile in any vertebrate species. Since the clock gene is believed to be involved in photoperiodic responses, including timing of spring phenological events in birds^50,51^, positive assortative mating for methylation may result from an effect of methylation at this *locus* on timing of arrival from migration and pre-breeding activities, favoring the pairing between individuals with synchronized life cycles. An alternative interpretation is that similarity in methylation during the breeding season arises as an effect of synchrony in breeding activities, therefore being the consequence, rather than the cause, of synchronization of spring life-cycle events.

### Methylation in relation to sex and life stage

We found clear evidence for variation in methylation in relation to age and sex. At clock poly-Q and timeless, nestlings had higher methylation than older individuals and females had higher methylation than males. The pattern was more complex and partly reversed for clock 5′-UTR, as methylation was lower in nestlings than in adults among females but not males, and was lower in female than male nestlings whereas no difference existed at the adult life stage.

We are aware of two cases where age-related variation in methylation in birds has been studied in the wild. Methylation at the avian glucocorticoid receptor gene did not differ between nestling and adult superb starlings (*Lamprotornis superbus*) whereas methylation at the agouti-related neuropeptide varied with age in the black grouse^23,36^. In humans, DNA methylation changes over the lifespan, but predominantly in the first year of life and throughout later childhood and adolescence, becoming more stable in the adulthood^52^. As a general pattern, repetitive elements are usually heavily methylated and become hypomethylated with age; gene promoters can be hypo- or hypermethylated according to their biological function and the presence of multiple methylated CpG sites in promoters causes stable silencing of genes^53^. At the mechanistic level, changes in the expression of enzymes regulating methylation could lead to generalized or gene-specific modulation of methylation with age^54^. The present age-related variation in methylation may contribute to cause the age-dependent variation in phenological traits, which is strongly expressed in barn swallows^48^.

Sex differences in DNA methylation have been studied in humans and other mammalian model organisms^55^, seldom in domestic birds^56^, while information on sex differences from the few studies of non-artificially selected avian models in the wild is extremely limited^36^. Barn swallows show variation in behaviors that may depend on circadian rhythmicity and photoperiodic responses according to sex, including for example timing of migration^37,57^. The sex-related differences that we observed in methylation may therefore partly account for such behavioral sex differences. In the present study, however, we did not investigate the environmental factors, if any, that could contribute to generate the observed variation of methylation levels according to age and sex, and very limited knowledge of methylation in wild vertebrates prevented us from speculating on which factors could in fact be responsible for such age- and sex-dependency.

### Within-individual consistency in methylation at the nestling and adult stage

The DNA methylation landscape is dynamically patterned during development and also at later life stages^58^, implying that methylation can change during life, as present results suggest to be the case at four *loci* (see above). However, individuals that at the nestling stage had high methylation also did so at the 1-year old stage at four of the six *loci* that we investigated, implying that *relative* methylation levels tended to remain constant across individuals during the first year of life. Consistency of methylation level has been previously documented during the first life stages (e.g. from childhood to first pregnancy) in humans^59^. The present results thus suggest that individual consistency in behavioral patterns that is observed at phenological or other behavioral traits that may depend on circadian clocks, can partly result from consistency of individual methylation level during life^47,48^. Cry1 and per3 represented an exception to the positive relationship between methylation levels at different life stages, with cry1 even showing a negative relationship between life stages. However, the mechanism and the function, if any, of such negative relationship for cry1 remains obscure at present.

### Methylation in parents and offspring

The results from the mother-offspring resemblance analyses clearly showed high resemblance at the nestling stage but such associations almost completely vanished as offspring reached the adult stage. Mother-offspring resemblance could arise via shared-environment effects. Additional, not necessarily mutually exclusive mechanisms that can induce resemblance in methylation are via the maternal germline, genetically-based variation in susceptibility to methylation and/or early maternal effects mediated by egg quality.

Interestingly, for per2 we found evidence for differential resemblance of maternal methylation levels with methylation of offspring of either sex. This result is consistent with observations of offspring sex-specific inheritance of methylation levels of either parent in ICR (Imprinting Control Regions)^60^.

Extra-pair fertilizations afforded an opportunity to indirectly test for environmental effects on father-offspring resemblance in methylation. The results, in this respect, were complex and only partly conclusive. For clock poly-Q there was a significant paternity by paternal methylation interaction effect on offspring methylation at the nestling stage, conclusively showing that resemblance with the offspring was larger for genetic that for non-genetic fathers. Hence, for clock poly-Q there was evidence for inheritance of methylation levels independent of environmental effects. Such father-offspring resemblance could arise because of transmission of paternal methylation levels via germinal cells and/or via genetic effects on susceptibility to methylation in prenatal or early postnatal life stages. Inheritance of methylation levels at clock poly-Q can have major ecological consequences because in the barn swallow methylation at clock poly-Q predicts variation at spring phenological traits^37^. Father- (and mother-) offspring resemblance in methylation could possibly contribute to establish parent-offspring resemblance in phenology traits.

For the other five *loci*, the interaction effect between paternity and paternal methylation at the nestling stage was either marginally non-significant after FDR correction (clock 5-UTR), or far from statistical significance (remaining *loci*), implying no statistically significant difference in the slopes of the relationships between methylation of the offspring and that of their genetic compared to non-genetic, social fathers. However, a closer inspection of the relationships within either paternity group suggested that the relationships between offspring and genetic-fathers at some *loci* had larger associated effect sizes (*t* values) than those between offspring and social fathers at the nestling or at the adult stages of the offspring. Albeit non-conclusive, these results may thus suggest father-offspring resemblance of methylation levels independent of environmental effects also at the other *loci*, besides clock poly-Q. In fact, the relationships between methylation of the non-genetic, social fathers and the offspring that they attended were not statistically significant. However, for clock poly-Q the relationship between methylation of the offspring at the nestling stage and that of their social father was negative, rather than null or positive as expected in the case of no or, respectively, non-null environmental effects. The interpretation of the latter result is open to speculation. Because the genetic father-offspring relationship was positive, whereas the relationship was negative for the social father, it might be speculated that mothers generating extra-pair offspring were fertilized by extra-pair males whose methylation level was negatively correlated with that of their mate. The function of such differential assortative mating^34^ with the social mate compared to the extra-pair mate might consist in increasing the epigenetic diversity of the offspring, with potential advantages in terms of adaptation to unpredictable ecological conditions, in line with the theory that considers the function of extra-pair fertilizations as a mean to increase phenotypic diversity of the offspring^61–63^.

Overall, the parent-offspring resemblance analyses suggested that genetic parents of either sex can differentially contribute to determine offspring methylation at individual *loci* and at different offspring life stages. Genetic fathers, in particular, seemed to contribute to offspring methylation at some *loci* of the clock genes also at the adult stage, whereas this was not the case for genetic mothers. In addition, the effect of methylation of the social (non-genetic) father on methylation of the extra-pair offspring was very weak, suggesting only minor, if any, effects of shared environment between fathers and offspring on resemblance in methylation.

### Methylation and breeding date

Genetic polymorphism at clock has been shown to be associated with phenological variation in several, though not all, vertebrate species tested so far^50,64–66^, including the barn swallow^51^. Besides genetic polymorphism, however, methylation state may also intervene in regulating clock-dependent photoperiodic responses. In a previous study we showed that methylation at the clock poly-Q predicted timing of breeding of old female barn swallows from the same geographical area where the present study was carried out^37^. To assess the robustness of the previously reported relationship, here we considerably expanded the sample size of the previous study and confirmed that large methylation at clock poly-Q is associated with early breeding in females but not in males. The relationship between breeding date and methylation at clock poly-Q held for both yearling (not tested in a previous study^37^) and older individuals, which are known to breed on average at different times^48^. As previously noted^37^, the sign of the relationship between methylation at clock poly-Q and breeding date is consistent with the expectation. This is the case because increasing methylation levels are expected to reduce the expression of CLOCK/BMAL1 transcription factor, thus reducing the transcriptional activation of the clock genes.

In addition, the present analyses on an expanded sample disclosed a marginally non-significant (P = 0.062) negative association between breeding date, independently of sex and age, and methylation at the clock 5′-UTR *locus*, which was not previously detected^37^, suggesting that methylation at other regions of clock may also be functionally relevant to timing of breeding. However, we did not find any hint for an association between methylation at other four circadian clock genes and breeding date in both sexes, which were not tested in the previous study.

Populations of birds and other organisms have undergone rapid phenotypic changes in phenological traits during the last decades, likely in response to climate change^67^, which have occurred at a pace that may be difficult to explain by invoking relatively slow micro-evolutionary processes^67^. The observed association between methylation at clock and breeding date (present study) or migration dates^37^, and the observation of parent-offspring resemblance in methylation at this gene may help explaining rapid phenological change in the barn swallow and, potentially, also in several other bird species^67,68^. This is the case because methylation at clock poly-Q, which shows high resemblance between genetic parents and young offspring, may mediate phenological shifts at least in early life stages, with likely carry-over consequences in adulthood, independently of any micro-evolutionary change in population genetic composition at clock.

### Concluding remarks

In conclusion, we found extensive evidence for differential variation in methylation levels according to sex and age, whose physiolocal and ecological causes remain to be elucidated. In addition, we also showed extensive, yet *locus*-dependent parent-offspring resemblance in methylation levels, with a hint that, for fathers, resemblance with the offspring is more strongly mediated by variation in germline methylation, genetic variation in susceptibility to methylation or sperm factors rather than by extrinsic environmental factors during early life stages. Such intergenerational resemblance in methylation has obvious implications for estimates of genetic heritability of traits that depend on clock genes. There is widespread evidence that rapid phenological changes have occurred in animal populations in response to climate change, and the fast pace of such changes has proven difficult to be reconciled with micro-evolutionary changes in populations. Epigenetic effects can rapidly generate phenotypic plasticity in phenological and other traits. The association between methylation at clock and phenological traits, in combination with intergenerational resemblance in methylation levels can thus provide a mechanistic basis to interpret the rapid phenological changes that populations have recently undergone in response to climate change.

## Methods

In spring 1999–2002 and 2010–2016 we captured all adult breeding barn swallows at a total of 12 colonies (=farms) located near Milan (Northern Italy) over nine years. Swallows were sexed, individually marked, subjected to blood sampling for methylation and genetic parentage analyses, and released. The breeding birds were assigned to their nest by observation of color rings. The nests were inspected every 2–5 days. When nestlings were 6–12 days old, they were also individually marked and a blood sample was collected for methylation and genetic paternity analyses. In all years, all adults at the breeding colonies were captured to identify the marked offspring born in the previous year that were recruited as 1-year-old breeding adults. These recruits were also subject to blood sampling for methylation analyses. Thus, for each breeding pair that generated one (or more; see below) recruited offspring, we measured methylation for the male and the female mates, and for their offspring both as nestlings in the year of birth and in the year following that of birth, when they reached sexual maturity.

Because in the years preceding those of blood sampling for methylation analyses all breeding adults were captured in the study colonies and barn swallows have extremely high breeding philopatry, in each sampling year we could assume that adult individuals that had not been captured in the previous year were ca. 1-year-old individuals, immigrating from other colonies (unless they were local recruits)^41^. Thus, in each year, breeding parents could be assigned to either of two age classes: yearling (1-year old) or ‘older’ (two or more years old). All the recruits thus were in the ‘yearling’ age class.

Breeding date (expressed as Julian date of laying of the first egg in the first clutch) was known for all the parents and also for part of their offspring that were later recruited as breeders.

### Paternity analyses

Genetic paternity analyses allowed us to assess if the parental father of the recruited offspring was its biological (hereafter ‘genetic’) father or it rather was the non-genetic (hereafter ‘social’) father because the offspring was sired by another male.

Genetic paternity analyses were performed according to previously published protocols^41,45^. Full methodological details for paternity analyses are reported in the Supplementary Material (SOM).

### Methylation analyses

We analyzed a total of six *loci* at five clock genes: clock, per2, per3, cry1, and timeless. When we started the present work no barn swallow genome sequence was available. We therefore decided to exploit the synteny between passerine birds to isolate the barn swallow genomic sequences of interest. Mainly, the genomes of *Ficedula albicollis*, *Phylloscopus trochilus* and *Pseudopodoces humilis*, were used. Except for clock, where PCR primers for the poly-Q exon and 5′-UTR region of the gene, specific for barn swallow, were already available^69^, we concentrated our attention on the promoter region of the other selected genes. A 5,000 bp region upstream of the predicted ATG start codon was identified in the different species, sequences were aligned and the portions conserved in all species were examined to identify candidate CpG methylation sites, flanked by DNA sequences suitable for PCR primer design. For each specific gene the primers were designed to cover the greatest possible number of CpG sites within the promoter region, taking into account the necessary length of the PCR amplicon, length of the target sequence, and primers that avoided CpGs. Sequences were designed using PyroMark Assay Design software (Qiagen, Germany). The identified primers were used to amplify the homologous sequence present in the barn swallow genome. Those fragments were purified (Wizard SV Gel and PCR clean-up System, Promega, Madison, Wi, USA) and sequenced (BMR genomics, Padua, Italy) to confirm their homology and to design specific PCR primers required for bisulfite pyrosequencing. Primer sequences are reported in SOM (Table 2S).

One µg DNA (concentration 50 ng/µl) was treated using EZ DNA Methylation-Gold™ Kit (Zymo Research, Orange, CA, USA) according to the manufacturer’s protocol. Final elution was performed with 30 µl of M-Elution Buffer. Bisulfite-treated DNA was stored at −20 °C and used shortly after treatment. Analysis of DNA methylation was performed using previously published methods^37,69^, with minor modifications. Briefly, a 50 µl PCR was carried out in 25 µl of GoTaq Green Master mix (Promega, Madison, WI, USA), 1 pmol of the forward primer, 1 pmol of the biotinylated reverse primer, 50 ng of bisulfite-treated genomic DNA and water. The biotin-labelled primers were used to purify the final PCR product using Sepharose beads. The PCR product was bound to Streptavidin Sepharose HP (Amersham Biosciences, Uppsala, Sweden) and the Sepharose beads containing the immobilized PCR product were purified, washed, denatured using a 0.2 M NaOH solution, and washed again using the Pyrosequencing Vacuum Prep Tool (Pyrosequencing, Inc., Westborough, MA), as recommended by the manufacturer. Then, 0.3 µΜ pyrosequencing primer was annealed to the purified single-stranded PCR product and pyrosequencing was performed using the PyroMark MD System (Pyrosequencing, Inc.). The degree of methylation was expressed as percentage of methylated cytosines divided by the sum of methylated and unmethylated cytosines (%5mC). Every sample was measured three times and the average of the replicates was used in statistical analyses. Assays which did not result in good quality pyrograms were repeated. If after 3 replicates the pyrogram quality was still not satisfactory according to pyrosequencing quality controls, the measure resulted in a missing value (see below and SOM for sample sizes). Each assay also included a bisulfite conversion check to verify full conversion of the DNA.

The mean coefficients of variation of methylation levels for the different *loci* was: clock 5′-UTR: 1.0%; clock poly-Q: 1.3%; cry1: 18.5%; per2: 1.0%; per3: 19%; timeless: 1.0%. The frequency distributions of methylation levels at the six *loci* is reported in Fig. 1S in SOM

### Statistical analyses

The complete version with full details of the Statistical analyses section is reported in the SOM. For clarity, the description of the statistical analyses is here organized so to correspond to the main sub-sections of the *Results*.

Because methylation variables are proportions, dependent methylation variables were always arcsin√x-transformed.

#### Methylation in pair members

We tested if methylation was correlated between males and females forming the same social breeding pair in linear mixed models (LMM) assuming a Gaussian error distribution with methylation of females as the dependent variable, methylation of the male mates as the independent variable, and year and colony as random factors.

#### Methylation in relation to sex and life stage

Variation in methylation according to sex and life stage was also analyzed in LMMs. For each *locus*, each methylation datum of parents and offspring was classified according to sex (male: 1; female: 2) and life stage (parent and 1-year-old offspring: 1; offspring at the nestling stage: 2). In the models, methylation was considered as the dependent variable, sex and life stage as fixed effect factors (with their two-way interaction), and year and colony as random effects. In addition, we also included a random factor family, i.e. a factor where the father (whether genetic or social), the mother, and their offspring at both the nestling and the adult stage were assigned the same code. The effect of the sex by life stage interaction is always presented in the *Results*, but when it was non-significant it was excluded from the model including the main effects. In these analyses, we also tested for the (random) effect of year and colony on methylation, by comparing the model including both random effects with reduced models that contained only the year or, respectively, the colony effects, by likelihood ratio tests.

#### Methylation at the nestling and 1-year-old recruit stage

The relationship between methylation at the 1-year-old stage (dependent variable) and at the nestling stage (independent variable) was analyzed in LMMs with year and colony as random effects.

#### Methylation in parents and offspring

The relationship between methylation at the nestling stage or at the 1-year-old recruit stage and methylation of the father and mother was analyzed in LMMs where we included methylation of the nestling as the dependent variable and methylation of the father and of the mother as independent variables. In addition, we included a factor ‘paternity’ to account for the fact that the father was the genetic or, respectively, the social, non-genetic father of the offspring. The interaction between paternity and methylation of the father allowed us to test whether the relationship between nestling’s and father’s methylation differed between genetic and social fathers. Finally, we also included a factor offspring sex to account for variation in methylation between male and female offspring and its interaction with maternal methylation, to account for any differential effect of methylation of mothers on methylation of sons as compared to daughters. The interaction between paternal methylation and offspring sex was not tested because of admixture of genetic and social fathers (see also SOM). The effect of sex as well as the effect of the interaction between paternity and paternal methylation were removed from final models when statistically non-significant. However, even when the paternity by father’s methylation was statistically non-significant, we still reported and inspected the within-paternity group relationships because these could provide information as to whether the *strength* (not the *slope*) of the relationship between nestling and father differed according to paternity (see also SOM).

As an exception to the above modelling approach, because of very high collinearity between mates at clock 5′-UTR (r = 0.648, n = 64, P < 0.0001; see also *Results*), for this *locus* the relationships with the mother or the father were tested in separate models.

#### Breeding date in relation to methylation

We tested whether methylation at the six *loci* statistically predicted breeding date. We thus designed LMM with sex and age (yearling vs older) as fixed effects and methylation, and their two-way interactions as predictors. In the models we also included year as a random factor and, in addition, a factor ‘family’ which accounted for the fact that in the dataset pairs of mates and, in some cases, trios (two parents plus one breeding offspring) were included. In the analyses on the effect of methylation at the two clock *loci*, where we also considered information on methylation and breeding date already reported in a study based in both Italy and Switzerland^37^, we also included a random factor ‘study area’ (Italy or Switzerland). Because the mean and the variances of the clock 5′-UTR and clock poly-Q between the present methylation data and those previously reported^37^ differed, methylation data were standardized to a mean of 0 and a variance of 1 within the two datasets.

Because repeated tests were run on the same individuals at the six *loci*, the results of the statistical tests were corrected according to the false discovery rate (FDR) procedure (see also SOM). Throughout the *Results*, uncorrected P values are presented and those that remained significant after FDR correction are marked with a ‘^†^’.

The analyses were run using SAS 9.3 statistical package.

#### Sample sizes

We sampled 58 pairs of breeding adults, and their recruited offspring both as nestlings and as 1-year-old recruits. Of these breeding pairs, 5 had two recruited offspring and 1 had three recruits included in the sample (65 offspring in total). The 65 offspring comprised 54 males (39 WPO and 15 EPO) and 11 females (8 WPO and 3 EPO). This sex-bias in recruitment is typical of the barn swallow and other birds due to female-biased natal dispersal^47^.

The size of the sample for each parental sex, life stage and individual *locus*, after exclusion of the outliers (i.e. observations that deviated more than 3 standard deviations from the mean), is reported in details in the SOM.

Information on breeding date was available also for for 37 offspring that were recruited as yearling breeders. In addition, we considered breeding date and methylation data at the two clock* loci* for 58 males and 26 females that were already included in a previous study^37^.

The Regione Lombardia administration gave permission for this study (permits no. 2959 and 11316; see also *Compliance with guidelines and regulations* section at the bottom of the main text).

All experiments were performed in accordance with relevant guidelines and regulations. All experimental protocols were approved by the Regione Lombardia administration (permits no. 2959 and 11316) following approval by the Istituto Superiore per la Protezione e la Ricerca Ambientale (ISPRA).

## Supplementary information

Supplementary Online Material

## Data Availability

The dataset supporting the conclusions of this article will be made available by the corresponding author upon request.
