# Supplementary material for: Inter-generational resemblance of methylation levels at circadian genes and associations with phenology in the barn swallow
Source: Sci Rep. 2019 Apr 24;9:6505. doi: 10.1038/s41598-019-42798-3 (PMC6482194; doi:10.1038/s41598-019-42798-3)
Supplement: Supplementary file 1 — Supplementary Online Material [file 41598_2019_42798_MOESM1_ESM.docx]

***Supplementary Online Material***

***for***

**Inter-generational resemblance of methylation levels at circadian genes and associations with phenology in the barn swallow**

*Nicola Saino^1^, Benedetta Albetti^2^, Roberto Ambrosini^1^, Manuela Caprioli^1^, Alessandra Costanzo^1^, Jacopo Mariani^2^, Marco Parolini^1^, Andrea Romano^1,3^, Diego Rubolini^1^, Giulio Formenti^1^, Luca Gianfranceschi^4^, Valentina Bollati^2^*

*Introduction - Mechanisms of action of ‘clock’ genes*

In vertebrates, circadian rhythms are regulated by a set of ‘clock’ genes. In both birds and mammals, the suprachiasmatic nucleus (SCN) contains a molecular circadian oscillator, which is entrained by light information^42^. In birds, the SCN, the eyes and the pineal jointly perform circadian pacemaker functions, regulating daily timing in physiology and behavior. Circadian rhythms at the cellular level are maintained by molecular oscillations via transcription/translation feedback loops. Although the photoperiodic information is differently integrated in birds and mammals, in both classes, the circadian timing system is also essential for measuring changes in day length, which then triggers seasonal neuroendocrine responses and synchronize circannual rhythms. In essence, the transcription/translation feedback loops depend on transcriptional activators, CLOCK and BMAL1, which drive the expression of transcriptional repressors, period 1 (per1), period 2 (per2), period 3 (per3), timeless (tim) and the cryptochromes cryptochrome 1 (cry1) and cryptochrome 2 (cry2), which generate negative feedback onto the activators. Unlike mammals, birds do not express a per1 and have been shown to express only per2 and per3^43–47^.

*Paternity analyses*

Genetic parentage analyses were performed according to Costanzo et al. (2017 a,b). Briefly, DNA was extracted by alkaline lysis from blood samples and diluted to a final concentration of 50 ng/µL (Saino et al., 2008). Genotyping of nestlings and adults was performed in a single multiplex reaction on a total of five loci: Adcyap1, POMC, Hir7, Hir17, Hir20. Either forward or reverse primers were fluorescently labelled. Polymorphism was determined using a commercial fragment analysis service (Macrogen Inc., Seoul, Republic of Korea) (Bazzi et al., 2015). GeneMarker® version 2.4.2 software (Softgenetics) was used to score fragment lengths of each individual. Cervus version 3.0.3 software was used to perform parentage assignment. The presence of extra-pair paternity was defined when the genetic father identified by parent pair analyses differed from the social father identified during behavioural observations. We did not find any case of brood parasitism (i.e. all nestling genetically matched with the social mother).

*Statistical analyses*

Because methylation variables are proportions, dependent methylation variables were always arcsin√x-transformed.

*Methylation in pair members.* We tested if methylation was correlated between males and females forming the same social breeding pair in linear mixed models (LMM) assuming a Gaussian error distribution with methylation of females as the dependent variable, methylation of the male mates as the independent variable, and year and colony as random factors. Because the nature (dependent or independent) of the methylation variables of either sex cannot be established unequivocally, we also repeated the analyses by including methylation of males as the dependent variable and methylation of females as the independent variable, again while including year and colony as random effects. We could not apply the more appropriate bivariate linear mixed modelling (Dingemanse and Dotchermann, 2013) because such models either failed to converge or produced unreliable parameter estimates. However, because at all *loci* either LMM led to reciprocally similar results, we only present the LMM with female methylation as the dependent variable.

*Methylation in relation to sex and life-stage.* Variation in methylation according to sex and life-stage was also analyzed in LMMs. For each locus, each methylation datum of parents and offspring was classified according to sex (male: 1; female: 2) and life stage (parent and 1-year-old offspring: 1; offspring at the nestling stage: 2). In the models, methylation was considered as the dependent variable, sex and life-stage as fixed effect factors (with their two-way interaction), and year and colony as random effects. In addition, we also included a random factor family, i.e. a factor where the father (whether genetic or social), the mother, and their offspring at both the nestling and the adult stage were assigned the same code. The effect of the sex by life-stage interaction is always presented in the *Results*, and its effect is excluded from the model including the main effects only when it was non-significant. In these analyses, we also tested for the (random) effect of year and colony on methylation, by comparing the model including both random effects with reduced models that contained only the year or, respectively, the colony effects, by likelihood ratio tests.

*Methylation at the nestling and 1-year-old recruit stage.* The relationship between methylation at the 1-year-old stage (dependent variable) and methylation at the nestling stage (independent variable) was analyzed in LMMs with year and colony as random effects.

*Methylation in parents and offspring.* The relationship between methylation at the nestling stage or at the 1-year-old recruit stage and methylation of the father and mother was analyzed in LMMs where we included methylation of the nestling as the dependent variable and methylation of the father and of the mother as independent variables. In addition, we included a factor ‘paternity’ to account for the fact that the father was the genetic or, respectively, the social, non-genetic father of the nestling. The interaction between paternity and methylation of the father allowed us to test whether the relationship between nestling’s and father’s methylation differed between genetic and social fathers. Finally, we also included a factor offspring sex to account for variation in methylation between male and female offspring and its interaction with maternal methylation, to account for any differential effect of methylation of mothers on methylation of sons as compared to daughters. The interaction between paternal methylation and offspring sex was not tested because of admixture of genetic and social fathers in the sample. However, an analysis restricted to within-pair offspring alone disclosed non-significant interaction effects between paternal methylation and offspring sex on offspring methylation both at the nestling and the adult stage (details available upon request). The effect of sex as well as the effect of the interaction between paternity and paternal methylation were removed from final models when statistically non-significant. However, when the paternity by father’s methylation was statistically non-significant, we still reported and inspected the within-paternity group relationships because these could provide information as to whether the *strength* (not the *slope*) of the relationship between nestling and father differed according to paternity. Indeed, the paternity by father’s methylation interaction term tests whether the slopes of the relationship differs according to paternity but does not provide conclusive information as to the relative *strength* of the relationship. This is the case because two relationships can have the same slope but still differ in *strength* of the association (i.e. the effect size).

As an exception to the above modelling approach, because of very high collinearity between methylation of the father and of the mother at *clock* 5’-UTR (r = 0.648, n = 64, P < 0.0001; see also *Results*), for this locus the relationships with the mother or the father were tested in separate models. For the other loci, no collinearity issue emerged (correlation in methylation between mates: unsigned r value < 0.29).

*Breeding date in relation to methylation*. We tested whether methylation at the six *loci* statistically predicted breeding date. We thus designed LMM with sex and age (two-levels fixed effect factors) and methylation, and their two-way interactions as predictors. In the models we also included year as a random factor and, in addition, a factor ‘family’ which accounted for the fact that in the dataset pairs of mates and, in some cases, trios (two parents plus one breeding offspring) were included. In the analyses on the effect of methylation at the two *clock* loci, where we also considered information on methylation and breeding date already reported in Saino et al. (2017) from a study in both Italy and Switzerland, we also included a random factor ‘study area’ (Italy or Switzerland) to account for the geographical origin of the data. Because the mean and the variances of the *clock* 5’-UTR and *clock* poly-Q between the present methylation data and those reported in Saino et al. (2017) differed, methylation data were standardized to a mean of 0 and a variance of 1 within the two datasets using all the methylation data available.

Because repeated tests were run on the same individuals at the six *loci*, the results of the statistical tests were corrected according to the false discovery rate (FDR) procedure. For example, in the analyses of the effect of sex and life-stage, the false discovery rate (FDR) procedure for the effect of life-stage was applied to the six P values associated with this effect. We did not apply FDR correction to the within-paternity group analysis of the association between parent and offspring methylation to avoid too large sacrificial loss of power of the tests. In addition, we did not apply FDR correction to the test of the relationship between breeding date and methylation at *clock* because these were run on a much larger set of individuals than the tests run for the other *loci*. Throughout the *Results*, uncorrected P values are presented and those that remained significant after FDR correction are marked with a ‘†’.

*Sample sizes*. We sampled 58 pairs of breeding adults, and their recruited offspring both as nestlings and as 1-year-old recruits. Of these pairs, 5 had two recruited offspring and 1 had three recruits included in the sample (65 offspring in total). The 65 offspring comprised 54 males (39 WPO and 15 EPO) and 11 females (8 WPO and 3 EPO). This sex-bias in recruitment is typical of the barn swallow and other birds due to female-biased natal dispersal (Møller 1994).

Overall, methylation data were available for (locus: no. of fathers, no. of mothers, no. recruits as nestlings, no. of 1-year-old recruits): c*lock* 5’-UTR: 58, 58, 65, 65; c*lock* poly-Q: 58, 58, 65, 65; c*ry1*: 57, 57, 57, 59; *per2*: 58, 58, 65, 65; *per3*: 58, 56, 65, 65; *timeless*: 44, 51, 51, 48. Some methylation data however appeared as outliers. After excluding the outliers, defined as the data that deviated by more than ± 3 standard deviations from the group (father, mother, nestling or, respectively, 1-year-old recruit) mean, the size of the samples was reduced to c*lock* 5’-UTR: 57, 58, 65, 65; c*lock* poly-Q: 56, 57, 64, 65; *cry1*: 57, 56, 57, 59; *per2*: 55, 57, 63, 63; *per3*: 58, 56, 64, 65; *timeless*: 43, 51, 50, 47. Thus, 18 out of 1406 (= 1.28%) methylation data points were excluded as outliers.

Information on breeding date was available for the 65 mothers and fathers included in the sample and also for 37 of their 65 offspring that were recruited as yearling breeders. In addition, we considered breeding date and methylation data at the two c*lock* *loci* for 58 males and 26 females that were already included in a previous study (Saino et al. 2017).

**Table 1S. Methylation of adult female parent in relation to methylation of the male mate.** Linear mixed models of methylation of females at the six *loci* in relation to methylation of the male mate. In the models, we included year and colony as random effects.

___________________________________________________________________________

F df P coefficient (SE)

___________________________________________________________________________

Clock 5’-UTR (n = 57)

Methylation of male mate 14.75 1,39 <0.001† 0.31 (0.08)

Clock poly-Q (n = 55)

Methylation of male mate 1.31 1,37 0.259 0.13 (0.12)

Cry1 (n = 55)

Methylation of male mate 3.99 1,37 0.053 -0.80 (0.40)

Per2 (n = 54)

Methylation of male mate 2.27 1,36 0.140 -0.17 (0.11)

Per3 (n = 56)

Methylation of male mate 0.29 1,38 0.596 -0.16 (0.29)

Timeless (n = 37)

Methylation of male mate 0.97 1,20 0.337 -0.06 (0.06)

__________________________________________________________________________

†: Significant after FDR correction

Table 2S. Sequences of the primers used in the present study.

| **Region of**  **interest** | **Primers** | **Sequence analysed** | **PCR annealing temperature** |
| --- | --- | --- | --- |
| 5'UTR | **F**: 5'-Bio-GGAGGATTAGGAGTAAATGAAA-3'  **R**: 5'-CATAACATATCACACTTTAATC-3'  **Seq**: 5'-CTTTAATCTTATAATAAAC-3' | 5′-AAG**C/TG**TACAAAT-3′ | 50.8 |
| Poly-Q exon | **F**: 5'-Bio-GTTAGTAGTTTGTAATAAAATTTG-3'  **R**: 5'-CTATCACCACCTATCCCATA-3'  **Seq**: 5'-CATTACTACTCCACATACTAC-3' | 5′-TTGTTA**C/TG**GTTTTA-3′ | 49.5 |
| CRY1 | **F**: 5'GGTGTAAGAGGGATTATTTTAATT-3'  **R:** 5'-Bio-CCTCCTAAAATCCCTTCCAA-3'  **Seq:** 5'-GGGGTTTTTTTTGATTAATA-3' | 5′-**C/TG**TTT**C/TG**A**C/TG**GTAT**C/TG**GG-3′ | 52 |
|  |  |  |  |
| PER2 | **F**: 5'-GGTAGTAAGGAAAGTGGGTTTTTT-3'  **R:** 5'-Bio-TTTCCTAAATCTAATTACTCACCA-3'  **Seq**: 5'-GTTTGTATAGAGAGTAAGAG-3' | 5′-TGA**C/TG**ATTTTTGTTTATTAGTAAAAAAAGTTTTTT**C/TG**GATTT-3′ | 50 |
| PER3 | **F:** 5'-ATTTGGGATTGGTATTTGAAGTAG-3'  **R:** 5'-Bio-CTAACACTCCAAACAACCAACTC-3'  **Seq:** 5'-CAACACAATATCACTCTTTT-3' | 5′-AAC**A/G**TTACC**A/G**CCACTCTTTAATACTACTT-3′ | 55 |
| TIMELESS | **F**: 5'- GAGGAGTTAAGGGATGAAGGAG-3'  **R:** 5'-Bio-TACTCCCCCATTCCCTCC-3'  **Seq**: 5'-CCAACACCCCAAATC-3' | 5′-**A/G**CCCCTC**A/G**TTATCCTTTCAC-3′ | 64 |

Figure 1S. Frequency distribution of methylation values at the six focal loci after exclusion of outliers (see Methods). The two extreme values for timeless are not outliers within their sex by life stage group.
